# The long-term impact of coronavirus disease 2019 on environmental health: a review study of the bi-directional effect

**DOI:** 10.1186/s42269-023-01007-y

**Published:** 2023-03-01

**Authors:** Prasenjit Chakraborty, Randhir Kumar, Sanjay Karn, Ankit Kumar Srivastava, Priya Mondal

**Affiliations:** 1 Department of Biosciences, School of Science, Indrashil University, Rajpur-Kadi, Mehsana, Gujarat 382740 India; 2 Laboratory of Cell Biology, National Cancer Institute, National Institute of Health, Bethesda, MD 20892 USA

**Keywords:** COVID-19, Environment, Sustainability, SARS-CoV-2

## Abstract

**Background:**

When health systems worldwide grapple with the coronavirus disease 2019 (COVID-19) pandemic, its effect on the global environment is also a significant consideration factor. It is a two-way process where the pre-COVID climate factors influenced the landscape in which the disease proliferates globally and the consequences of the pandemic on our surroundings. The environmental health disparities will also have a long-lasting effect on public health response.

**Main body:**

The ongoing research on the novel coronavirus severe acute respiratory syndrome coronavirus 2 (SARS-CoV-2) and COVID-19 must also include the role of environmental factors in the process of infection and the differential severity of the disease. Studies have shown that the virus has created positive and negative ramifications on the world environment, especially in countries most critically affected by the pandemic. Contingency measures to slow down the virus, such as self-distancing and lockdowns have shown improvements in air, water, and noise quality with a concomitant decrease in greenhouse gas emissions. On the other hand, biohazard waste management is a cause for concern that can result in negative effects on planetary health. At the peak of the infection, most attention has been diverted to the medical aspects of the pandemic. Gradually, policymakers must shift their focus to social and economic avenues, environmental development, and sustainability.

**Conclusion:**

The COVID-19 pandemic has profoundly impacted the environment, both directly and indirectly. On the one hand, the sudden halt in economic and industrial activities led to a decrease in air and water pollution, as well as a reduction in greenhouse gas emissions. On the other hand, the increased use of single-use plastics and a surge in e-commerce activities have had negative effects on the environment. As we move forward, we must consider the pandemic's long-term impacts on the environment and work toward a more sustainable future that balances economic growth and environmental protection. The study shall update the readers on the various facets of the interaction between this pandemic and environmental health with model development for long-term sustainability.

**Graphic Abstract:**

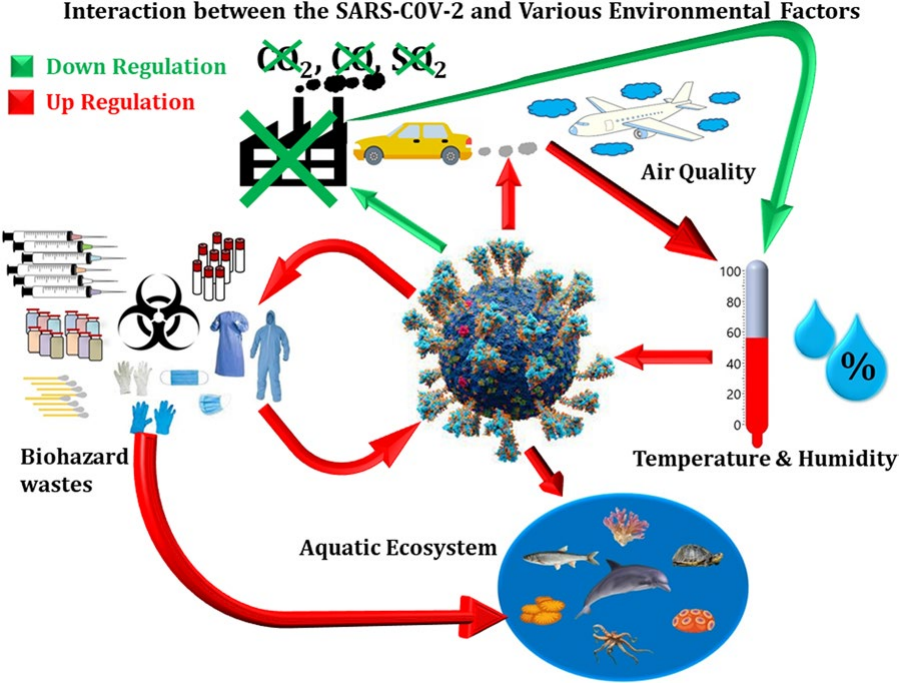

## Background

In early December 2019, an unusual pneumonia outbreak in Wuhan city, China, created an unprecedented and irreversible impact on the entire world 2/24/2023 12:58:00 PM. The symptoms of COVID-19 range from mild to severe, and include mainly fever, cough, and respiratory distress. Severe cases of pneumonia and hypoxemia result in considerable mortality (Rothan and Byrareddy 2020). The etiology has been linked to the new strain of coronavirus (SARS-CoV-2). According to the COVID-19 weekly epidemiological update by the World Health Organization (WHO), as of 9 May 2021, there have been a total of 163,965,336 COVID-19 cases with 3,397,147 fatalities (World Health Organization 2021). However, the new dynamics of the outbreaks seem highly variable among countries (Paiva et al. 2021).

Irrespective of the country, social distancing and mass testing have been the two major policies for checking the spread of the virus, till we had vaccines. The primary priority is to safeguard people's health. Lockdowns have been enforced, which lead to a reduction in Nitrogen Dioxide (NO_2_) and Particulate Matter with a diameter less than 2.5 µm (PM 2.5) concentrations in several European countries (Zambrano-Monserrate et al. 2020). In the Chinese province of Hubei, the implementation of strong social distancing measures was made in late 2019, affecting the country's main economic activities by shutting down the power plant and industrial facilities and halting their production. As a result, the use of vehicles has significantly decreased leading to a dramatic reduction in NO_2_ and PM 2.5 in important Chinese cities. According to Liu et al., greenhouse gas (GHG) emissions have dropped to proportions higher than in World War II or previous economic recessions (Liu et al. 2020). The USA contributed the most, with approximately a 13% decrease in its emissions, compared to the global decrease of 8.8%. A study in the State of Rio de Janeiro, Brazil, has observed a strong correlation between the different weather factors and the spread of COVID-19 (Rosario et al. 2020). Among all the different factors that were studied, solar radiation was found to be the most critical. It is very difficult to maintain a balance between reducing the severity of the disease and its devastating effect on the economy at large (Anderson et al. 2020). There are some general factors like the virus itself, its incubation time, and its effect on the susceptible population, which are similar in different geographical locations (Coccia 2020). On the other hand, different environmental factors such as air pollution, temperature, and humidity are specific for a particular location and individual, thus determining transmission dynamics. The transmission and survival of viruses responsible for respiratory diseases, such as influenza and SARS, are already known to be influenced by environmental media (Tamerius et al. 2013; Muthuraman and Lakshminarayanan 2021). So, it is of pivotal importance to know the role of environmental conditions in the transmission of the virus to raise awareness on the prevention of disease spread.

**Fig. 1:**
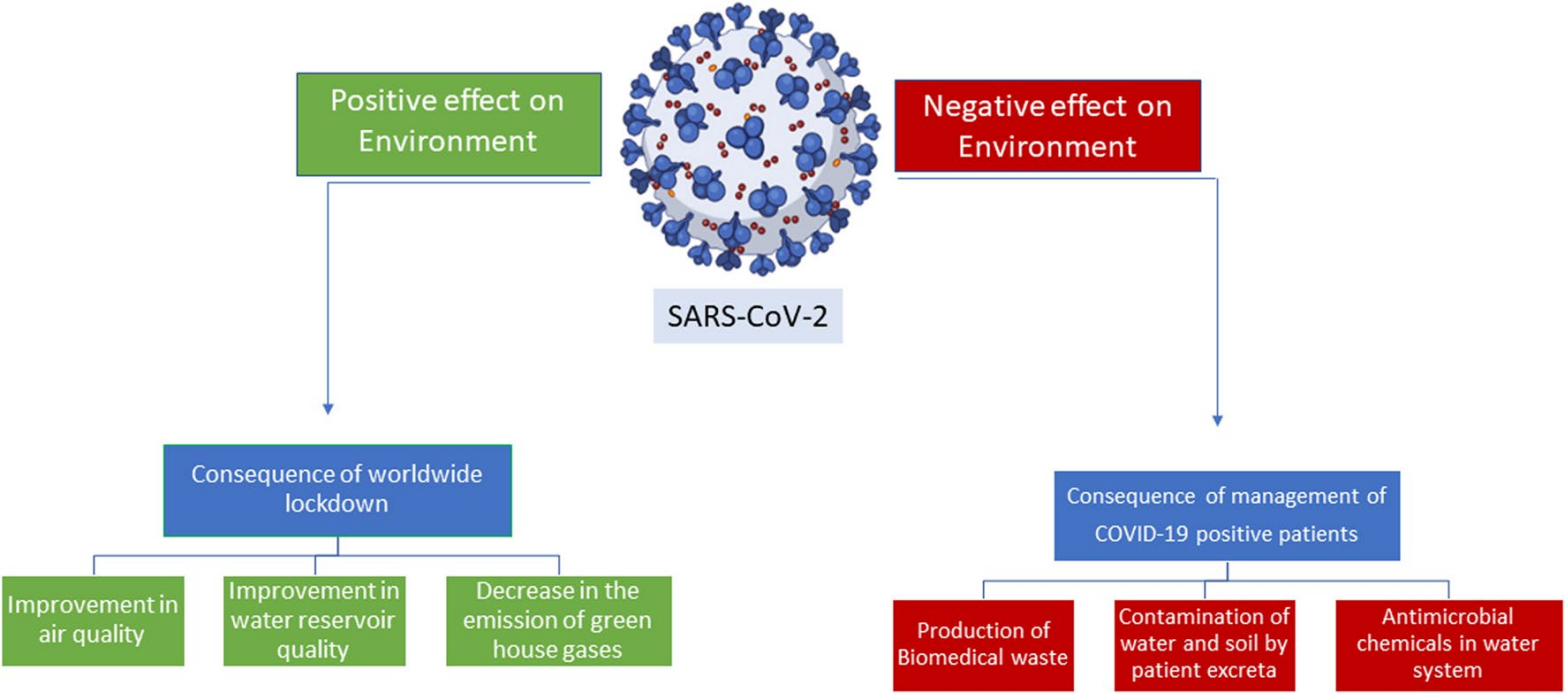
A flowchart depicting the long-term positive and negative effects of SARS-COV-2 on the environment

Another challenge to consider will be the enormous shift in biological waste composition and its management during and after the pandemic (Klemeš et al. 2020). A highly dynamic waste management system will be required to address the potential consequences of the pandemic. The environment thus plays a significant role in the transmission, pathogenesis, severity, and related morbidity caused by COVID-19. The effect of this pandemic will be felt on planetary health including climate change, biodiversity, urban built environment, and sustainability. The methodology for this study involved conducting a comprehensive literature review of published articles and reports on the long-term impact of COVID-19 on environmental health. The search for relevant articles was conducted using multiple databases, including PubMed, Google Scholar, Scopus, and ScienceDirect by providing different combinations of keywords. Inclusion criteria for the articles included in the review were peer-reviewed articles published in English and related to the topic of COVID-19 and its impact on environmental health. Exclusion criteria were articles that were not relevant to the topic or did not meet the quality standards for peer-reviewed publications. The articles were analyzed for their content and key findings, and the information was synthesized to develop a thorough understanding of the bi-directional impact of COVID-19 on environmental health. A narrative synthesis approach was used to identify patterns and themes in the data, and to provide a clear and concise overview of the findings. The bi-directional effect of COVID-19 on the environment in the long term at large has been summarized in Fig. 1. In this review article, we are going to discuss the different perspectives on environmental factors, encompassing climatic and anthropological parameters that have direct or indirect effects on the spreading and transmission of COVID-19 and sustainable development.

## Main text

### Role of temperature and humidity in the transmission of SARS-CoV-2

The temperature and its variations are predicted to have a profound effect on death from respiratory diseases (Tan et al. 2005; Pinheiro et al. 2014). Metz and Finn have reviewed a hypothesis that low absolute humidity supports the spread of influenza in temperate climates (Metz and Finn 2015). There are some other studies, which show that temperature modulates COVID-19 transmission through its effect on various meteorological factors (Oliveiros et al. 2020; Wang et al. 2020). Apart from providing helpful conditions for virus survival and spreading, low temperatures and humidity also hinder the innate immune response (Kudo et al. 2019; Sun et al. 2020). Cold conditions reduce the supply of blood immune cells to the nasal mucosa. Low relative humidity can reduce the capacity of cilia cells in the airway to remove virus particles. In addition, low-humidity environments impair the innate immune defense system, making it vulnerable to virus invasion. For the above-mentioned reasons, several studies are being dedicated to delineating the effect of temperature and humidity on the spread of COVID-19 with contradictory conclusions.

In the year 2020, Wu et al. employed a log-linear generalized additive model (GAM) to analyze the effects of temperature and humidity on COVID-19 infection and deaths in 166 countries (Wu et al. 2020). Considering both the lag and cumulative effects, a negative correlation has been discovered between temperature and relative humidity to that of daily new cases and deaths caused by SARS-CoV-2. Diurnal temperature range (DTR), on the other hand, is having a positive association with daily mortality of COVID-19 (Ma et al. 2020). In general, tropical climate delays the onset of confirmed COVID-19-positive cases in comparison to tempered climates (Méndez-Arriaga 2020). On the contrary, using wavelet transform coherence, and partial and multiple wavelet coherence, Iqbal et al. observed that the average daily temperature between 3 °C and 21 °C does not have any significant effect on the containment of COVID-19 in Wuhan (Iqbal et al. 2020). Meteorological data from 122 cities in China showed no evidence that COVID-19 cases would reduce in warmer weather (Xie and Zhu 2020). At < 3 °C, a 1 °C rise in the mean temperature was associated with a 4.861% increase in the daily-confirmed cases. Another study conducted in China does not support the hypothesis that a rise in temperature or ultraviolet radiation can reduce the transmissibility of the coronavirus (Yao et al. 2020). Outside of China, in the USA, Bashir et al. analyzed the effect of different climate indicators on the COVID-19 pandemic (Bashir et al. 2020). Although they found a significant association between average temperature, minimum temperature, and air quality with the spread of the disease, no evidence was obtained to suggest that warm weather suppresses the virus.

One probable reason for these contradictions could be that different studies were conducted with distinct data sets. Moreover, the population size and density were also different. As a result of which the probability of infection also varies between different regions. Research conducted at a global scale with a larger temperature and humidity range may provide an accurate correlation between these climatic factors and the spread of SARS-CoV-2. Another challenge will be the normalization of the complex epidemiological data generated from different parts of the world. Nonetheless, there seems to be a collective effect of temperature and humidity on the spread as well as the mortality caused by COVID-19.

### Air quality and COVID-19

Air pollution, especially urban air pollution can increase the rate of infection and mortality associated with COVID-19 pathogenesis (Liang et al. 2020; Zhu et al. 2020). An increase in pollutants such as nitrogen dioxide (NO_2_) per interquartile range has a significant effect on the disease severity and outcome. One of the reasons for this could be that the extent of lung damage the virus can cause depends on how much the respiratory system of the infected individual has already been compromised due to air pollution. Inhalation of excessive particulate matter, oxidants, and chemical pollutants can attenuate the immune system and decrease the ability of the lungs to clear pathogens residing in it (Qu et al. 2020). Many age-old infectious diseases are re-emerging due to global urbanization and accompanying climate changes (Weiss and McMichael 2004; Heymann 2005). Even short-term exposure to particulate matter with an aerodynamic diameter of both 10 and 2.5 µM (PM_10_ and PM_2.5_) has been found to enhance cardiovascular and respiratory mortality (Liu et al. 2019). During the 2002–2004 SARS outbreak in mainland China, a direct correlation was reported between case fatality and air pollution (Cui et al. 2003). A recent study with COVID-19 patients in southern California has found that air pollution may increase the severity and mortality associated with the disease (Chen et al. 2022).

It has been established beyond doubt that increases in air pollution cause a concomitant increase in acute COVID-19 infection. A saving grace in this regard is that a highly transmissible virus-like SARS-CoV-2 forces authorities around the world to declare total lockdown. Restricted human activities due to the lockdown helped reduce the pollution level in the atmosphere. In a study conducted in twenty-two cities in India, it was found that the harmful PM_2.5_ levels were significantly reduced in most regions (Sharma et al. 2020). A related study in a northwestern US city showed a positive impact of COVID-19 in decreasing the ultrafine particles present in the air (Xiang et al. 2020). The public health measures enforced to control the coronavirus outbreak resulted in a significant reduction in the NO_2_ column across China, South Korea, the US, and some western European countries (Bauwens et al. 2020). Nitrogen oxides are the chief catalysts of ozone formation in the troposphere. All these data cumulatively make an exemplary point to the environmental regulatory bodies that the air quality of our planet could be improved by the proper execution of stringent control measures.

### COVID and the aquatic ecosystem

The use of protective gear during COVID-19 all over the globe has tremendously increased the demand for single-use plastics and as a result, it polluted our aquatic ecosystem too. As per the report from the United Nations environment program, 150 million metric tons of plastic waste are presently in our oceans with an additional 13 million tons per year (UNEP 2020). Furthermore, due to the outbreak of COVID-19, the use of plastics in the manufacturing of protective gear has increased many folds. As a result, plastic pollution in the aquatic ecosystem has increased tremendously in the last 3 years. Studies from various parts of the world evidenced the occurrences of protective equipment near the coastline, beaches, and coastal cities which are polluting the aquatic ecosystem (Okuku et al. 2021; De-la-Torre et al. 2021). The increased occurrences of plastic waste in oceans can be due to the mismanagement of solid waste which may seep out into rivers and then finally into the oceans. COVID-19 protective gears are made up of polymers such as polyethylene, terephthalate, polyvinyl alcohol, polyvinyl chloride, polypropylene, and polystyrene, which may be seen floating as well as deposited on the sea floor as per the characteristics of the materials used in their preparation. In oceans, these plastic materials are broken down into microplastics under the action of UV radiation and wave action, concomitantly resulting in permanent pollution of the marine ecosystem (Henderson and Green 2020). These biomedical plastic wastes as well as microplastics can provide a good habitat for certain harmful and pathogenic microorganisms, leading to conditions like the acidification of oceans. They can act as vectors for different toxic pollutants, and harmful chemicals, as well as pathogens for antibiotic resistance genes resulting in an adverse effect on aquatic biodiversity (Wu et al. 2019; Li et al. 2020; Chen et al. 2020; Harvey et al. 2020; Eder et al. 2021; Lee et al. 2021). Reports from various researchers documented the adverse effect of COVID-19 protective gears waste on aquatic organisms, such as hermit crabs, puffer fish, octopuses, and aquatic birds like swans, mallards, and gulls (Jagiello et al. 2019; Lavers et al. 2020; Hiemstra et al. 2021). The biomedical waste of COVID-19 thrown into aquatic bodies can impact the swimming ability of aquatic animals, and their feeding habits and may lead to severe wounds, infections, and hypoxia-like conditions. These conditions will increase in nearby future concerning aquatic animals owing to a large number of plastic waste disposals in aquifers. To overcome these problems in the coming era, scientists, various active environmentalists, and policymakers should make advanced strategies for the use of natural and biodegradable materials for the preparation of single-use health care gear and proper solid waste management systems.

### Generation of biohazardous waste materials and measures to deal with it

Management of biomedical waste has assumed great significance in light of the rapid upsurge of COVID-19 infection. Around 15% of healthcare-related waste can be classified as hazardous because of its infectivity, toxicity, and/or radioactivity. According to the WHO, unsafe injections account for a significant proportion of new viral infections (Pépin et al. 2014). In India especially, there has been a huge expulsion of biomedical waste due to the pandemic caused by the novel coronavirus (Ramteke and Sahu 2020). Biomedical wastes which are used in managing the control of infections in society such as cotton swabs, syringes, blood bags, face shields, gloves, masks, PPE, and shoe covers are used in large amounts worldwide. This leads to an increase in the daily generation of biomedical waste by 5500 metric tons which was around 200,000 metric tons annually worldwide (Minoglou et al. 2017; Ranjan et al. 2020). In Wuhan city alone approximately 214 metric tons of additional biomedical waste were generated per day (Almulhim et al. 2021). Moreover, the mass vaccination campaign to fight COVID-19 has increased biohazard waste in terms of consumable waste products like empty vials, syringes, and needles (Hasija et al. 2022; Baumann et al. 2022).

Poor management of waste materials generated from a highly contagious virus with a relatively long survival period such as SARS-CoV-2 can cause immeasurable damage to the environment. Furthermore, due to mass vaccination around the globe, rampant uses of preventive gear against COVID were used. It has resulted in a surplus of biomedical waste at these vaccination centers. Consequentially, an increase in production, packaging, deep freezing, and transportation to fulfill the demand for a large number of vaccines resulted in heightened greenhouse gas emissions. This is evident in a report from Kurzweil et al. stating that 1100 kg of CO_2_ emission was released into the environment through one million doses of two mRNA vaccines (Kurzweil et al. 2021). It is a well-known fact that increases in greenhouse gas are primarily responsible for raising the temperature of the earth and causing heat waves across different parts of the globe. The emission of various chemicals such as dioxins and furans from the incineration of biohazard waste can cause an undesirable impact on the environment. It can lead to an increase in the overall particulate matter present within the atmosphere, which results in adverse health effects.

To prevent the unintended release of biological hazards like antibiotic-resistant microbes into the environment, proper handling of wastes generated from healthcare activities is necessary (Nzediegwu and Chang 2020; Das et al. 2021). Safety measures have been implemented across the world to minimize the waste generated by this pandemic. Despite these measures, the amount of healthcare waste being accumulated is a cause for concern. The life cycle of plastic waste products related to PPE kits and other healthcare accessories has been impacted due to the pandemic. The footprint of these waste products needs to be captured through new directions in the research and policies (Klemeš et al. 2020). Dumping of solid wastes in landfills can exacerbate the spread of the virus within the local community. Proper sorting, disinfection, and recycling of healthcare wastes through sustainable management may help reduce the spread of SARS-CoV-2. This in turn will safeguard the health of patients, doctors, nurses, and other healthcare staff along with the general population.

## Discussion

Since World War II, the coronavirus disease 2019, or COVID-19 pandemic as we know it is one of the greatest challenges faced by humanity. The global outbreak adversely affected the socioeconomic status of people worldwide (World Health Organization 2020). It disturbed the lives and livelihoods of many peoples globally. The ready availability of antivirals and vaccines against pandemic potential RNA viruses can prevent us from similar disasters in the future. Recently, some promising strides have been made in the development of such antivirals and vaccines (Saiz 2020; Pardi and Weissman 2020; Chakraborty et al. 2021). Massive hospitalizations, deaths, and socioeconomic damages notwithstanding, the pandemic have resulted in prodigious changes within the environment, and some of them have been on the positive side. Curtailment in social gatherings and significant reduction in financial activities have improved the quality of air, water, and other aspects of the environment (Rume and Islam 2020). A noticeable decrease in human interference and over-exploitation of natural resources has been observed during the pandemic. This indicates a unique reformation of human interaction with the environment which was associated with some short-term widespread effects on the improvement in air quality, cleaner water system, reduction in noise levels, and activity of wildlife animals (Diffenbaugh et al. 2020; Laughner et al. 2021). Apart from these positive impacts, some negative effects have also emerged such as poverty, food security, mental illness, and an increase in the amount of some pollutants due to specific measures implemented for the treatment of COVID-19-positive patients and to limit the spread of the virus.

Indeed, the situation of COVID-19 was a dreadful experience for human beings all over the world, but it taught us several possible ways that could be helpful to achieve global environmental sustainability. The sudden increase in hospitalization due to infection of COVID-19 also raises a question mark on the hospital management of many countries, especially in developing and underdeveloped countries. In India, a shortage of several medical facilities such as medical professionals, ventilators, medical oxygen, and antiviral drugs has been observed during the second wave of COVID-19 (Bhuyan 2021; The Lancet 2021). Hence, this pandemic taught the government and policymakers to change their old facility to cope with unexpected viral/medical emergencies in the future. During the COVID-19 pandemic, the production of biomedical waste increased significantly, adversely affecting the environment. The waste management systems of developing and underdeveloped countries were already strained (Srivastava et al. 2015). Hence generation of huge hospital waste made a new challenge to this country for their remediation. Many developing countries such as India are not able to manage COVID-associated waste efficiently making them more prone to face the consequential problems of waste disaster. It might increase the possibility of food chain contamination. So, it is very essential to develop a more efficient, modified, optimized, and automated waste management system to overcome any adverse environmental challenge in the future (Kothari et al. 2021; Kuppusamy et al. 2022). Its necessity became more prominent in the case of emerging viruses, whose mutational behavior and potential hosts are unknown.

**Fig. 2:**
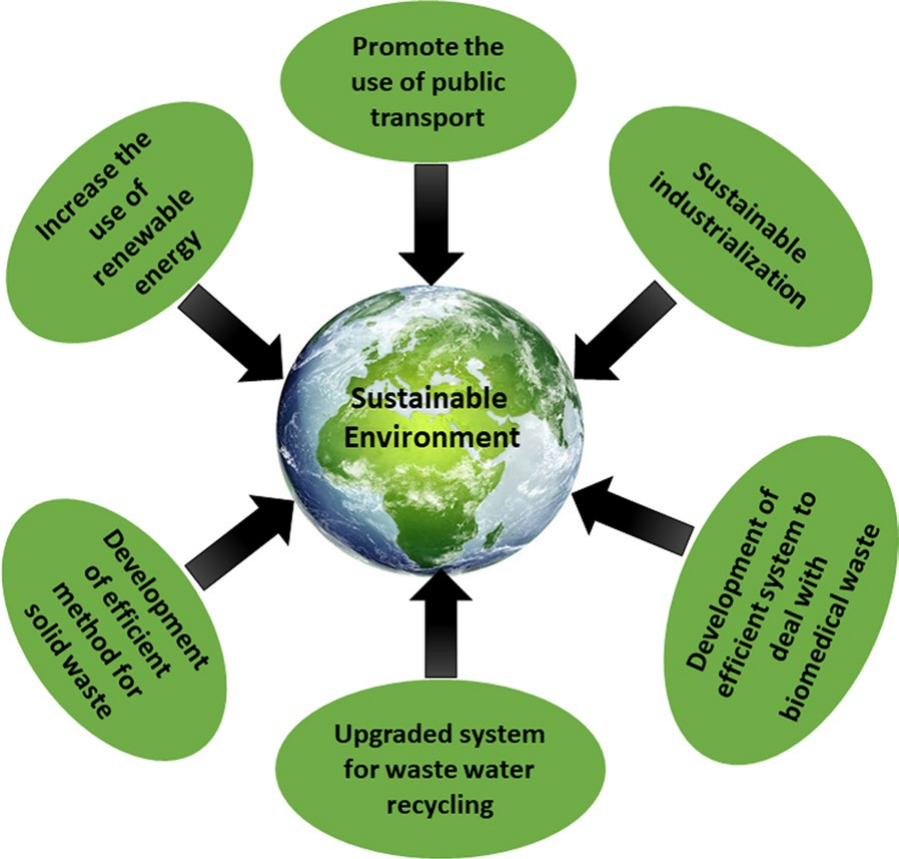
A model for sustainable development of the environment in the post-covid era

The current COVID-19 situation will certainly provide us with a novel testbed to understand the role of individual human beings and policymakers in conserving a sustainable environment (Fig. 2). This test bed could be helpful to answer many questions about the interrelation between the degradation of the global environment and the economic growth of a country. This could reveal the concept of sustainable industrialization and management of different solid waste to conserve the green ecosystem. Now it appears that the world economy is slowly returning and is expected to hit the pre-pandemic level very soon. Improvement in the world economy is appreciable but not at the cost of global climate change. So, it could be possible to make a suitable model based on results and analysis of the testbed that can define the feasible ways of ecologically responsible industrialization and personal behavioral changes in daily life, which support the development of a sustainable environment.

## Conclusions

In conclusion, this review study highlights the complex and bi-directional impact that the COVID-19 pandemic has had on environmental health. The pandemic has benefitted the environment by reducing air pollution levels and increasing water quality in some regions due to decreased human activity. On the contrary, the pandemic has also resulted in increased waste generation, particularly in the form of single-use plastics, and the accelerated shift toward online commerce. In addition, the pandemic has disrupted global supply chains, leading to increased deforestation and other environmental degradation. As the world is coming out of the devastating pandemic, it is important to consider the long-term environmental health effects and take steps to mitigate them. This may include increased investment in renewable energy, improvements in waste management systems, and shifts toward more sustainable consumption patterns. Additionally, governments and organizations can work together to create policies that promote environmental health and sustainability, such as regulations on plastic use and carbon emissions. Overall, the COVID-19 pandemic has underscored the interconnectedness of human health and environmental health and the need for continued efforts to protect both. As we work to recover from the pandemic and build a better future, it is critical that we prioritize the health of our planet and take action to ensure a sustainable and healthy world for future generations. The present study can provide a basis for the development of recommendations for future research in this area.

## Data Availability

Not applicable.

## Abbreviations

COVID-19: Coronavirus disease 2019
SARS-CoV-2: Severe acute respiratory syndrome coronavirus 2
GHG: Greenhouse gas
WHO: World Health Organization
NO_2_: Nitrogen dioxide
PM: Particulate matter
GAM: Generalized additive model
PPE: Personal protective equipment
